# Surviving to Thriving: A Grounded Theory of Post-Diagnostic Identity Reconstruction in Midlife and Older Autistic Adults

**DOI:** 10.1177/13623613261483304

**Published:** 2026-09-12

**Authors:** Jennifer A. Kirton, Angela Tod, Tony Ryan

**Affiliations:** 1 University of Liverpool, UK; 2 University of Sheffield, UK

**Keywords:** autism, late diagnosis, identity development, grounded theory, post-diagnostic support, midlife and older autistic adults, well-being, neurodiversity

## Abstract

This study explored how midlife and older autistic adults make sense of receiving an autism diagnosis later in life and how this shapes post-diagnostic identity reconstruction. Although rising numbers of adults are diagnosed in later life, little is known about how they interpret this experience or the factors shaping post-diagnostic adjustment. Constructivist grounded theory was used to examine experiences among 14 autistic midlife and older adults aged 50 to 73. Seventeen semi-structured interviews were analysed using iterative coding and constant comparison. An autistic advisory group supported reflexivity and analytic credibility. Diagnosis was valued but had a liminal effect. Two broad patterns of post-diagnostic experience were identified: *surviving*, characterised by self-blame and deficit-based interpretations; and *thriving, characterised by greater self-compassion, personal agency, and living in ways more aligned with their autistic identity.* Thriving was delayed and followed years of identity reconstruction. It unfolded through the *Thriving Cycle*, comprising three interconnected processes experienced as a dynamic and iterative. The *Thriving Cycle* illustrates how some midlife and older autistic adults moved beyond diagnostic recognition towards a more integrated and affirming autistic identity. These findings suggest that identity-focused support may help individuals move from ‘survival’ towards greater self-understanding, agency, and well-being.

**Lay Abstract:**

More adults are being diagnosed as autistic later in life, but little is known about how they make sense of this experience or how it affects their lives afterwards. This study explored how autistic adults diagnosed in midlife and older age understood their diagnosis and how it shaped the way they saw themselves. We interviewed 14 autistic adults aged 50 to 73. We explored their experiences of receiving an autism diagnosis later in life and what happened afterwards. An autistic advisory group helped ensure the findings reflected autistic perspectives. Participants valued receiving a diagnosis, but it also marked a period of uncertainty and adjustment. Two broad patterns of post-diagnostic experience were identified. Some participants remained in a pattern of surviving, marked by self-blame, and seeing themselves in negative ways. Others moved towards thriving, with greater self-compassion, a stronger sense of personal agency, and living in ways that were more consistent with their autistic identity. Thriving did not happen immediately after diagnosis but developed over time through years of making sense of their experiences. The study also identified the Thriving Cycle, a model describing three connected processes that supported thriving. These processes were experienced as ongoing, and people often moved back and forth between them over time. These findings suggest that receiving an autism diagnosis is only the beginning of the journey for many autistic adults. The Thriving Cycle shows how some midlife and older autistic adults moved beyond surviving towards thriving over time. The findings highlight the importance of providing post-diagnostic support to help autistic adults understand themselves and improve their well-being after diagnosis.

## Background

Increasing recognition of autism and improved diagnostic pathways have contributed to a rise in adults seeking assessment later in life (Russell et al., 2021). This has drawn attention to the ‘lost generation’ (Lai & Baron-Cohen, 2015) of autistic adults who grew up without their autism being recognised. Their needs were often missed due to narrow diagnostic criteria (Hull et al., 2017), gendered stereotypes in autism presentation that contributed to many autistic women and girls being overlooked in diagnostic systems (Bargiela et al., 2016; Hull et al., 2020), or long-standing patterns of masking and camouflaging (Hull et al., 2019). Many midlife and older autistic adults reach diagnosis only after decades of navigating misdiagnosis, chronic stress, and mental health difficulties without an explanatory framework (Crane et al., 2021; Jones et al., 2021; Lipinski et al., 2023).

Research increasingly suggests that receiving an autism diagnosis in adulthood can be beneficial. Reported outcomes include greater self-understanding (Leedham et al., 2020), improved self-compassion (Lewis, 2016), reduced self-blame (Lilley, 2021), and opportunities for identity development (Botha & Frost, 2020; Huws & Jones, 2010). Diagnosis may provide a coherent interpretive lens for previously misunderstood experiences and, when followed by appropriate support, can enhance well-being (Bargiela et al., 2016; Higgins et al., 2021; Stagg & Belcher, 2019).

However, these benefits are *not universal*. Much of the existing literature centres on individuals who are able to independently pursue self-education, community connection, and identity reconstruction post-diagnosis (Leedham et al., 2020; Tan, 2018), often in the absence of formal post-diagnostic support. At the same time, evidence indicates that such support is inconsistently available and frequently inaccessible for many autistic adults (Crane et al., 2020, 2021; Crowson et al., 2024).

Midlife and older autistic adults may face additional barriers: many have lived with decades of internalised stigma (Botha, 2021) and have relied on coping strategies developed without an understanding of autism (Milton & Sims, 2016). Long-standing patterns of unconscious masking and compensation, often established well before diagnosis and embedded as automatic behaviours, can further hinder adjustment (Hull et al., 2019; Livingston et al., 2020). Histories of mental health difficulties may also complicate the integration of a newly discovered identity (Mason et al., 2018). For these individuals, diagnosis alone may be insufficient to trigger meaningful change and enhance well-being.

Without structured support, many autistic adults describe the diagnostic process as incomplete or a transitional state in which diagnosis provides recognition but little practical guidance, often leaving individuals to make sense of their diagnosis alone (Crane et al., 2020; Lewis, 2016). This is particularly concerning because the post-diagnostic period is when many people require support to understand their experiences, acquire autism-related knowledge, and begin the process of identity reconstruction (Botha & Gillespie-Lynch, 2022). These challenges are compounded by structural barriers. In the United Kingdom, adult autism assessment often involves lengthy National Health Service (NHS) waiting lists (Macdonald et al., 2022), whereas private assessment can cost thousands of pounds, limiting accessibility (Jones et al., 2021). Even after diagnosis, access to funded post-diagnostic support remains inconsistent and frequently minimal (Crane et al., 2020; National Autistic Society, 2022). Consequently, many autistic adults complete a lengthy and costly diagnostic journey only to find that diagnosis alone does not automatically translate into improved well-being, identity integration, or meaningful support.

Collectively, these issues highlight that late-life diagnosis may initiate, but does not complete, the process of identity and well-being development for autistic adults. Diagnosis often provides initial awareness, yet identity integration typically requires further exploration, community connection, and meaning-making (Cage & Troxell-Whitman, 2019; Leedham et al., 2020). While some autistic adults can navigate this post-diagnostic journey independently through self-reflection and engagement with autistic communities (Huws & Jones, 2010; Tan, 2018), others require structured guidance, psychoeducation, or relational support to fully realise the benefits of diagnostic recognition (Botha & Gillespie-Lynch, 2022; Crane et al., 2020). Understanding these varied experiences is particularly important for midlife and older autistic adults, whose needs, histories, and support requirements differ from those diagnosed earlier in life (Mason et al., 2018; Wilson et al., 2019). However, despite growing recognition of the importance of post-diagnostic identity development, it remains unclear which external supports and personal resources best facilitate identity reconstruction and enable some autistic adults to experience more positive post-diagnostic outcomes than others.

Autistic identity has received increasing attention in recent research, with growing recognition of its complexity and its relevance for well-being (Botha, 2021; Cooper et al., 2021; Davies et al., 2024). Contemporary work conceptualises autistic identity as multidimensional (Davies et al., 2024), encompassing factors such as self-acceptance, identity development (Tan, 2018), and internalised stigma (Botha, 2021). Positive autistic identity, including acceptance and a sense of belonging, has been associated with improved well-being, whereas internalised stigma and camouflaging are linked to poorer outcomes (Botha & Frost, 2020; Hull et al., 2019; Livingston et al., 2020). Recent qualitative syntheses further highlight that identity development is closely tied to the process of diagnosis. Diagnosis acts as a catalyst, and identity development involves shifts in self-understanding, reappraisal of past experiences, increased connection with other autistic people, and movement from a deficit-based identity to a more positive identity (Nayyar et al., 2025). These developments sit within a broader paradigm shift in autism research, from deficit-based models towards neurodiversity-informed perspectives (Pellicano & den Houting, 2022). This shift is not only conceptual but also has practical implications, as the frameworks through which autism is understood, including clinical and support approaches, may shape how individuals interpret their diagnosis and develop their identity. Identity-affirming perspectives have been associated with improved self-esteem and well-being, whereas deficit-focused models may reinforce stigma and negative self-concept (Cooper et al., 2021). Recent oral history research has further highlighted the complexity of post-diagnostic identity reconstruction in late-diagnosed autistic adults, demonstrating processes of autobiographical reappraisal, identity renegotiation, and the development of shared autistic identity (Lilley et al., 2022, 2023). This work has been especially valuable in foregrounding autistic voices and demonstrating the depth and complexity of post-diagnostic meaning-making.

This study aimed to explore how autistic adults diagnosed in midlife and later life make sense of receiving an autism diagnosis and how processes of post-diagnostic identity reconstruction shape experiences of well-being over time. In this study, well-being is understood as a subjective and multidimensional construct, rather than simply the absence of psychological distress, and is considered within the broader social and relational context of autistic adults’ lives (Botha & Frost, 2020). Specifically, the study sought to understand the processes involved in post-diagnostic identity reconstruction and how these may shape post-diagnostic adjustment and well-being. In doing so, the study aimed to contribute to the growing literature seeking to understand identity reconstruction following autism diagnosis in midlife and later life.

## Method

### Ethical Considerations

Ethical approval was obtained from the University of Sheffield Research Ethics Committee, and all participants provided informed consent. The authors declare that they have no conflicts of interest.

All participants provided informed consent, were free to pause or stop interviews, and were offered debriefing information and signposting to support services. Particular care was taken given reports of distress associated with diagnosis and post-diagnostic experiences, consistent with recommendations for conducting ethical qualitative research with autistic adults (Nicolaidis et al., 2019).

### Design

This study adopted a constructivist grounded theory (CGT) methodology (Charmaz, 2014), chosen to explore how midlife and older autistic adults make sense of their diagnosis and its impact on well-being. CGT was selected because it foregrounds participants’ subjective meanings, supports co-construction of knowledge, and is well suited to examining identity-related processes.

### Participatory Methods Statement

This study was informed throughout by consultation with an autistic advisory group comprising four autistic adults with experience of receiving an autism diagnosis in adulthood. The advisory group contributed to the development and refinement of the interview schedule and advised on interview accessibility, communication preferences, pacing, sensory considerations, and participant-led interviewing approaches. They also supported interpretation of emerging findings, refinement of conceptual categories, and the development of the ‘Thriving Cycle’, while challenging deficit-based interpretations and advising on terminology and the relevance of findings to autistic lived experience. Their involvement enhanced the credibility, accessibility, and community relevance of the research. Following completion of the study, advisory group members were invited to become co-authors in recognition of their contributions but declined authorship and did not wish to be acknowledged individually in the manuscript.

### Positionality Statement

The first author identifies as neurodivergent, though not autistic, and brings prior professional and personal experience relating to autism, including sustained engagement with autistic communities and family experience of autism. Co-authors acted in a supervisory capacity. Their expertise included qualitative research, clinical practice, and medical research. Autism-specific lived experience expertise was provided by the autistic advisory group, whose contributions informed the study throughout. These experiences shaped the researcher’s interest in late-life autism diagnosis and sensitised them to issues relating to identity, stigma, and gaps in post-diagnostic support. Consistent with CGT, the authors acknowledge that knowledge was co-constructed through interaction between researcher and participants rather than discovered objectively.

### Participants and Recruitment

Fourteen autistic adults (eight women, six men) aged 50 to 73 took part, generating 17 interviews in total. Inclusion criteria were (a) aged ⩾50, (b) a formal autism diagnosis, and (c) ability to participate in an interview. Participants were recruited through multiple channels, including autism organisations, local autistic social groups, social media, university networks, and snowball sampling. The recruitment avenues were selected to reach individuals often underrepresented in autism research, particularly midlife and older adults who did not grow up with diagnostic recognition.

### Participants

Fourteen participants took part in the study, generating 17 interviews. Participants were predominantly White British (*n* = 13), with one White European participant, reflecting limited ethnic diversity. Eight participants were in paid employment, working across a range of sectors including academia, the NHS, public services, administration, charity, and other professional roles; five participants were retired, and one was unemployed. Six participants lived alone, four lived with a partner, and four lived with a partner and children or stepchildren. Seven participants were married or in a relationship at the time of the study. In terms of relationship history, seven participants had experienced divorce and five had never married. Nine participants had children and/or stepchildren, while five did not. (see Table 1 for participant characteristics).

**Table 1.** Demographics of Participants.

Participant pseudonym	Age (in years)	Sex	Age @ DX (in years)	Time since DX (in years)
Conceptualised as ‘Thriving’				
Theo^a^	53	M	49	4
Peter	55	M	48	7
Yvette	52	F	51	1
Gary	53	M	47	7
Sheila	50	F	42	8
Sophia	53	F	52	1
Emma	54	F	50	4
Conceptualised as ‘Surviving’				
Ralph	73	M	59	14
Christine^a^	58	F	52	6
Amy	52	F	48	4
James	62	M	54	8
Catrina	60	F	54	6
Sylvia	52	F	46	6
Walter^a^	65	M	57	8
Participants were predominantly White British (*n* = 13), White European (*n* = 1), with limited ethnic diversity across the sample.				

*Note*. Marital status. M = Married; N = Never married; D = Divorced.

a Denotes two interviews conducted.

### Data Collection

Semi-structured interviews were conducted by the first author. To accommodate sensory and communication preferences, participants were offered the opportunity to break the interview into different sessions if they wished and chose the format, and location and structure of their interview. Particular attention was given to how participants understood the impact of diagnosis on their sense of self, daily life, and overall well-being. A flexible, participant-led interviewing approach was used, with communication preferences, terminology, pacing, and sequencing adapted in response to individual needs. Participants were able to guide the depth and direction of discussion and could pause, skip questions, or redirect topics at any time. The semi-structured interview guide was developed iteratively, informed by existing literature on adult autism diagnosis, identity, and well-being, and designed in consultation with the autistic advisory group to ensure relevance, clarity, and accessibility. It was designed to explore key domains including life history, experiences of diagnosis, identity development, well-being, social relationships, coping strategies, and support needs. Open-ended questions were used alongside prompts to elicit elaboration and clarify meaning. The full interview schedule, including example prompts, is provided in the appendix. Member checking occurred throughout the interviews to support clarity, understanding, and co-construction of meaning.

Information regarding access to post-diagnostic support was also collected during interviews; none of the participants reported receiving formal post-diagnostic support following diagnosis. In total, 35 hr of interview data were recorded and transcribed verbatim. Interviews ranged from approximately 1.5 to 3.5 hr, with a mean duration of 2.5 hr (median ≈ 2.4 hr). In some cases, interviews were conducted across two (*n* = 3) sessions to reduce participant burden.

Interviews were conducted in a range of formats according to participant preference, including participants’ homes (*n* = 7), university settings (*n* = 4), a community venue (*n* = 1), a workplace (*n* = 1), and remote audio calls (*n* = 4). Most interviews were conducted face-to-face (*n* = 10), with four conducted remotely.

### Data Analysis

Data were analysed following Charmaz’s (2014) CGT procedures: initial line-by-line coding, focused coding, and constant comparison across interviews. Initial coding remained close to participants’ accounts, with codes compared continuously within and across transcripts. Focused coding was used to synthesise key patterns and develop more abstract categories. Diagramming was a central analytic tool, used throughout to map relationships between codes and categories, explore processes, and support the development of the emerging theoretical model. Memo-writing complemented this process by documenting analytic decisions, reflexive observations, assumptions, emotional responses, and the potential influence of the researcher’s positionality on data interpretation. Regular discussions with the autistic advisory group further supported reflexive analysis by challenging emerging interpretations and ensuring that conceptual categories remained grounded in autistic perspectives. The grounded theoretical model, termed the ‘Thriving Cycle’, was developed iteratively through constant comparison, diagramming, and discussion with the autistic advisory group. The conceptual categories of ‘surviving’ and ‘thriving’ were developed as interpretive theoretical constructs through the analytic process and were not presented to participants for validation or intended to represent participant self-identification. Analysis occurred alongside data collection, with theoretical saturation reached when no new properties of the categories were identified. NVivo was used to support data organisation only.

## Results

The results are presented in three stages. First, we describe the shared impact of receiving an autism diagnosis across the sample. We then examine two broad patterns of post-diagnostic experience, conceptualised as *surviving* and *thriving*. Finally, we present the Thriving Cycle (Figure 1), a grounded theoretical model developed through the analysis in this study, which explains the dynamic processes underpinning movement towards a thriving autistic identity.

**Figure 1.**
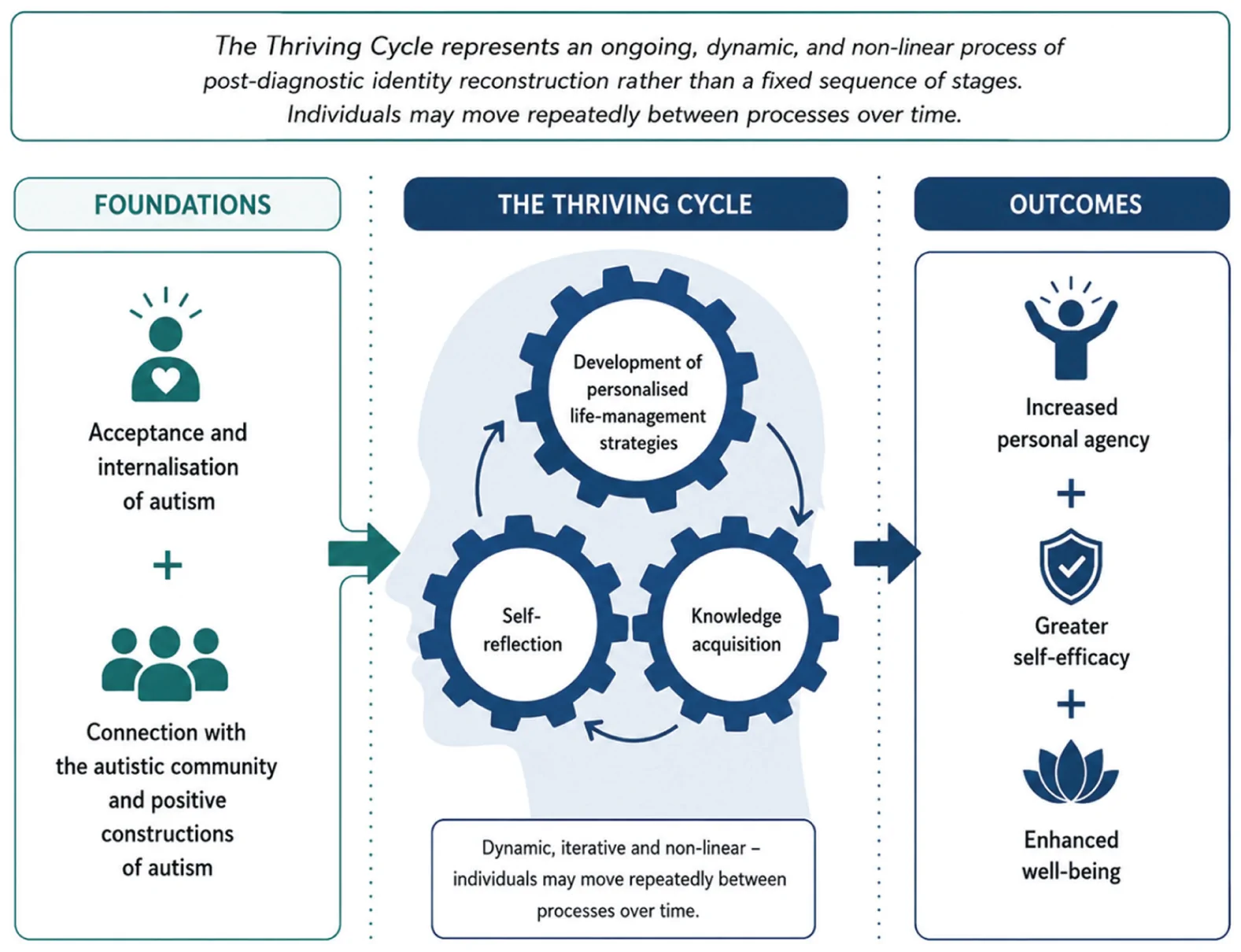
The Thriving Cycle: A grounded theoretical model of post-diagnostic identity reconstruction.

Quotations appear in italics and are labelled with pseudonym, sex, age, and years since diagnosis (e.g., Emma/F/54/4 = Emma, female, aged 54 years, 4 years post-diagnosis).

For all participants diagnosis represented a pivotal moment and, at the time of the interview, was universally regarded as beneficial. However, diagnosis alone had a largely liminal effect, providing recognition without necessarily resulting in immediate or sustained improvements in well-being. The analysis identified two broad patterns of post-diagnostic experience. One pattern reflected *surviving*, in which diagnosis was valued, but its impact remained limited and did not translate into substantial change. The second reflected *thriving*, characterised by fundamental shifts in participants’ understanding of themselves and the ways in which they lived their lives. Importantly, thriving was not an immediate outcome of diagnosis. Rather, participants whose accounts reflected the thriving pattern engaged in an ongoing process of self-discovery and identity reconstruction, conceptualised in this study as the Thriving Cycle. These patterns are not intended to represent fixed or mutually exclusive categories. Rather, they provide an analytic framework for understanding common patterns of post-diagnostic experience across the dataset. Participants’ accounts frequently contained elements of both patterns, suggesting movement between them over time and supporting a dynamic, nonlinear conceptualisation of post-diagnostic adjustment.

### Experiences of Diagnosis

Although all participants viewed diagnosis as a positive addition to their lives, their accounts demonstrated considerable variation in emotional reactions and subsequent adjustment. Diagnosis was described as valuable to ‘knowing’ oneself, offering a new lens through which to compare oneself with others, and deepening understanding of how autism shaped *relationships.* Participants also emphasised the significance of recognising that they were part of *a tribe* of other autistic people who were different like them. For some, however, initial reactions were marked by shock and grief:

My initial reaction, I suppose, was to think that there was something wrong with me and I was never going to be able to be normal or change things or improve things . . . I’ve heard people kick off when you describe it like this, but I would describe it as grieving . . . a grieving process for the person that I thought I was and the things I thought I was going to perhaps achieve. (Emma/F/54/4)

By contrast, other participants described the diagnosis as more immediately affirming.

I felt great, ‘cause I thought, God, against the odds, look where I am and, you know, what I’ve done and what I’ve achieved, you know. So it did, it felt amazing, it felt really good. (Yvette/F/52/1)

Across the sample, diagnosis facilitated reinterpretation of past experiences and promoted greater self-compassion and reduced self-blame. It provided an explanatory framework for long-standing social and sensory difficulties:

Once I got it [diagnosis] I started to put the puzzle pieces together and think this is why I was like that, this is what happened, I can see why I felt that I didn’t fit in, I can see why I hated going to parties . . . I felt like an outsider. (Christine/F/58/6)

Diagnosis also reshaped understandings of close relationships, allowing participants and significant others to re-evaluate interaction patterns and, in some cases, respond more supportively. Recognising that it was neither fair nor realistic to compare themselves directly with non-autistic peers, enabling them to reconsider previously internalised expectations of themselves.

I’m then [post diagnosis] in a position to kind of say, well actually, I can stop giving myself a hard time about that because it doesn’t matter how hard I tried there, I was never going to be able to do that because . . . It’s just beyond my ability to do . . . it’s that old quote, judge a gold fish by its ability to climb a tree and it’s going to be shit. I can’t do those things. They’re just not something that it’s in me. (Peter/M/55/7)

For others, diagnosis reduced self-blame and facilitated a more compassionate view of their own histories. However, as the following extract illustrates, post-diagnostic adjustment could still be protracted and distressing:

It was a bit mixed; it was nice to have that affirmation that, yes, I did have Asperger’s syndrome but then I’d say I was through crisis for about a year of coming to terms with it . . . I didn’t really feel like I had any support, apart from [spouse], obviously, but I didn’t know where to turn for support . . . for about a year, mentally, I think I was in turmoil. (Gary/M/53/7)

### Surviving: Diagnosis as Explanation Without Integration to Self

Participants in reflecting the ‘surviving’ pattern recognised benefits of diagnosis but did not progress to deeper integration of autism into their self-concept. For these participants, diagnosis primarily functioned as an explanation for past experiences but did not substantially shift how they felt about themselves.

It [diagnosis] helps me to understand me better because, I put things down to having autism now whereas before I just thought I was mad. (Sylvia/F/52/6)

They described focusing on the barriers they perceived autism created in their lives and what they perceived was wrong with them. Diagnosis often became a justification for perceived shortcomings, and autism was construed as a deficit, illness, or indication that there was something fundamentally wrong with them.

I knew then there was something wrong with me . . . I just don’t seem to realise these things which I should realise. (Ralph/M/73/14)

For some participants, diagnosis reinforced a sense of powerlessness, with autism understood as a fixed and *non-modifiable* condition rather than something that could be integrated into a more agentic sense of self:

If it’s a lifelong condition . . . I can’t really do anything about that. (Walter/M/57/8)

It [diagnosis] was sort of interesting . . . oh, this explains things. But then . . . a bit of a drop because . . . it’s a non-treatable condition, so I’m stuck. (James/M/62/8)

Surviving participants frequently described living in a near-constant state of stress, responding to crises without a sustainable repertoire of coping strategies and experiencing pronounced emotional ‘peaks and troughs’:

I feel like I’m hanging on by my fingertips over the abyss . . . From time to time, I fall into it. And then when I’m not in it, I’m always hoping I don’t fall into it again. It’s like a tightrope: I’m constantly on a tightrope. . . . I try not to think about it ‘cause it is terrifying. (Catrina/F/60/6)

Participants often talked about how exhausting life was, that they felt they had to work harder than non-autistic peers to survive:

I’ve never considered suicide or anything like that but sometimes I just feel like being alive is too tiring . . . it is hard work and I don’t think anybody who hasn’t got autism or Asperger’s, I don’t think it’s easy for them to understand that . . . Sometimes, life, I just feel like I’ve had enough of life. (Gary/M/53/7)

Participants whose accounts reflected the surviving pattern also reported feeling profoundly unsupported by services, describing how they had ‘*fallen through the gaps’* (Catrina/F/60/6) as they were living independently and apparently coping. They felt that there was no structure or appropriate support to assist them in managing their diagnosis or daily life. They were left alone to struggle on and battle through inadequate service provision:

I have had a really bad experience with the mental health service, so I really haven’t got anything good to say about them because it’s been a slog, it’s been a fight all the time. I can’t see any positives that way. (Christine/F/58/6)

While surviving participants accepted diagnosis as an explanation for their differences, they struggled to accept themselves. This appeared to be underpinned by negative feedback from others that had been absorbed into participants’ self-concept, alongside experiences of self-criticism, shame, and, in some cases, explicit self-hate.

Especially when she [daughter] starts with the insults because obviously with my [low] self-esteem I do start to believe what she’s saying. (Amy/F/48/4)

Although many had contact with other autistic people through social groups, this contact was primarily for companionship and did not appear to be used as a source of autism-related knowledge or critical reflection on their own experiences.

Participant: I now have a peer group where I feel . . . where I felt accepted. That was very important.

Interviewer: So, when you heard people experiencing the same things as you, it made you feel better about experiencing them yourself perhaps?

P: No. [pauses] Well, I think it was more that I didn’t feel alone, I didn’t feel by myself anymore. (James/M/62/8)

### Thriving: Reconceptualising Self-Identity

In contrast, participants reflecting the ‘thriving’ pattern had actively reconceptualised their self-identity following diagnosis. They now explicitly rejected their earlier notions of brokenness or deficiency and embraced autism as central to who they were:

It is just this idea that you know a person with autism. I’m a person with a bag. I put the bag down, and I’m no longer a person with a bag. I am not going to put my autism down . . . because it’s who I am. (Peter/M/55/7)

Thriving participants reported that diagnosis validated their experiences and increased their confidence to organise their lives around their needs. Over time, they developed detailed insight into their own patterns of experience and identified key elements necessary to maintain their well-being:

It has taken me the last few years to come to terms with this diagnostic bombshell, gain understanding and start moving through a position of acceptance and now, celebration of my particular difference . . . (Sheila/F/50/8)

They described a strong sense of self-efficacy and agency, along with proactive strategies for managing daily life. The transition to a thriving autistic identity occurred at different time points and was not immediate; many described an initial period post-diagnosis of emotional upheaval, which often lasted several years:

Yeah, there was a lot of different emotions and things to process afterwards. Its only really now [four years post diagnosis] that I think that I’m starting to, because I can talk about it now without getting upset which I couldn’t do for a long time. (Emma/F/54/4)

I think there was a whole load of denial going on with the diagnosis, to be honest with you. But I think, you know, in this last couple of years or so [6 years post diagnosis], very much so reflecting, and thinking, ah yeah, that makes sense. In fact, it’s been a huge light bulb moment. (Sheila/F/50/8)

For thriving participants, post-diagnostic trajectories were characterised by personal growth, self-empowerment, and increased capacity to shape their lives in ways that aligned with their autistic identity.

### The Thriving Cycle: Acceptance, Positive Constructions, and Self-Discovery

A key distinction between the surviving and thriving patterns was participants’ engagement in processes of post-diagnostic identity reconstruction. Thriving participants engaged in a self-directed process of discovery and identity work, conceptualised as the *Thriving Cycle*. This process was initiated when participants accepted and internalised their autism diagnosis and was facilitated by exposure to positive constructions of autistic identity. Together, these elements enabled a deeper post-diagnostic journey of self-discovery.

Three interrelated and mutually reinforcing processes were identified as central to thriving. These did not occur in a fixed sequence but interacted dynamically, with participants moving between them over time.

1. Acceptance and internalisation of autism;
2. Connection with the autistic community and positive constructions of autism;
3. Engagement in an ongoing ‘thriving cycle’ of knowledge acquisition, reflection, and personalised life-management strategies.

### Acceptance and Internalisation of Autism

For thriving participants, acceptance and internalisation of autism was a gradual process, often extending over several years post-diagnosis. Exposure to strengths-focused understandings of autism acted as a key facilitator. These participants viewed autism as an intrinsic aspect of their identity rather than a deficit, and this perspective supported self-empowerment and a more affirming outlook:

It becomes part of you (Sheila/F/50/8)

Support from significant others, particularly partners, was also important. Being seen and accommodated as an autistic person reinforced self-acceptance and enabled further exploration of identity:

I always had mental health problems. But, we didn’t have any idea it was autism at the root of it . . . he [husband] wasn’t surprised. But he’s always seen me as a person, so . . . But, you know, he’s very up to learning about it, and sort of like, you know, he’s accommodating. (Sheila/F/50/8)

Participants who displayed thriving patterns increasingly interpreted challenges as arising from societal misunderstanding rather than personal failure. This mindset shift reduced self-blame and fostered confidence in being different:

You know what, you might have difficulties with that, but do you know what? That’s not the end of the world, you’re not a failure, you can do lots of things and you know just because that person there doesn’t think [the same way as you do], well they’re a dickhead! (Peter/M/55/7)

Acceptance of difference, rather than striving to be ‘normal’, allowed some participants to stop dwelling on miscommunications and to find greater peace in their relationships with others and themselves:

When I was beginning to accept myself . . . my biggest wish was I wish I knew how to stop doing this [upsetting people] but if I don’t know it how I can stop doing it and I never will be able to, so I said to myself ‘come on Sophia you’ve just got to accept yourself’ I’m a great believer in that. (Sophia/F/53/1)

For some, this level of internalisation took several years: self-acceptance and internalisation thus provided the foundation on which thriving was built.

### Connection With the Autistic Community and Positive Constructions of Autism

Participants reflecting the thriving pattern also engaged actively with affirming and empowering perspectives on autism. These positive constructions challenged earlier deficit-focused views and provided alternative narratives that emphasised difference and strength rather than pathology. Participants encountered such narratives in personal relationships, social media, literature, and online communities; the voices of other autistic people were particularly salient:

You don’t have to listen to the educationalists, you don’t have to listen to doctors, you don’t have to listen to psychiatrists or psychologists . . . Often it takes somebody from the same place [being autistic] . . . to say, you don’t have to listen to them. You just be you. (Peter/M/55/7)

Validation from other autistic people, especially in online spaces, helped participants feel less isolated and enabled them to contextualise their own experiences:

Twitter has been revelatory for me because it allows me to find out how other people are beginning to realise . . . I’m so far from alone with being a late diagnosed autistic. (Sophia/F/53/1)

Online communities also offered opportunities both to receive and provide support:

The biggest positive I’ve got from being on the internet groups, is that knowledge that there are other people out there like me. (Emma, F/54)

Through these engagements, participants moved away from deficit-based diagnostic language towards more empowering, identity-affirming narratives:

A positive thing was interacting with other people . . . recognition of what is different about me, so that I can build upon this for my own research. (Theo/M/55/4)

Hearing personal stories from other autistic people promoted validation, encouraged further exploration of their own autistic experience, and fostered a sense of possibility.

### Developing Agency: Towards a Thriving Autistic Identity

A defining characteristic of the thriving pattern was the development of personal agency. Participants described increasing confidence in understanding and advocating for their needs, making informed decisions about their lives, and implementing personalised strategies that supported well-being and authentic autistic identity.

Once participants had internalised their autism and engaged with positive constructions of autistic identity, they described making ongoing, purposeful changes to their lives. This iterative process, the ‘Thriving Cycle’ (see figure 1), involved autism knowledge acquisition, self-reflection, and the development of personalised life-management strategies. Participants reported increased confidence, self-efficacy, and empowerment, alongside enhanced overall well-being.

I think I’m becoming much more aware of my autism. But I’m also learning to befriend it and accommodate it more, and actually it gives me more strength in some ways now. (Yvette/F/52/1)

They no longer felt like passive passengers in life, reacting only to crises. Instead, they described actively directing their lives in ways that supported their own needs and priorities. Thriving participants reported a more positive self-image than those who were surviving. Meeting other autistic people validated participants’ experiences and enhanced their self-understanding. They emphasised the importance of self-care and no longer directly compared their experiences and achievements with non-autistic peers, instead anticipating that their trajectories would be different and accepting this as legitimate. Although difficulties and stressors persisted, participants felt better equipped to manage them. They described being more able to identify triggers for anxiety and to plan strategies for times of crisis.

I’m bloody good at my job actually, but some things I do, I realise they take more spoons [energy] . . . now I do things to make things better. Like [delivering] training takes a hell of a lot of energy from me. I love it, but it’s exhausting! So I just manage my diary. If I have training one day, I do nothing the night after and make sure my diary is light the next day, it works . . . So, I understand now how to best work things for me. (Peter/M/55/7)

The successful use of these strategies further reinforced their sense of self-efficacy. As a result, thriving participants were more likely to advocate for themselves and request reasonable adjustments from others.

I haven’t just survived; I now thrive and embrace the strengths that autism gives me. (Sheila/F/50/8)

## Discussion

This study explored how midlife and older autistic adults make sense of a late-life diagnosis and engage in post-diagnostic identity reconstruction over time. Well-being emerged as closely tied to these processes of self-understanding, identity integration, and agency. While prior research has identified both benefits and challenges associated with adult diagnosis, the present findings extend this work by offering a process-based explanation for why outcomes diverge. This process-based account builds on a growing body of literature that conceptualises post-diagnostic adjustment as ongoing, identity-focused, and shaped by access to social and informational resources (Davies et al., 2024; Kiehl et al., 2024; Nayyar et al., 2025). Specifically, the ‘Thriving Cycle’ provides a conceptual model linking identity reconstruction, access to positive autistic narratives, and the development of agency.

The study demonstrated that, while all the participants valued receiving an autism diagnosis, its impact alone was largely *liminal* rather than transformative. Diagnosis provided recognition, validation, and an explanatory framework for past experiences, yet did not automatically lead to improved well-being. Instead, two broad patterns of post-diagnostic experience emerged: ‘surviving’ post-diagnosis, experiencing limited or short-lived benefits; and ‘thriving’, but only after engaging in a sustained process of identity reconstruction, self-reflection, and knowledge acquisition. These findings support earlier evidence (Nayyar et al., 2025) that diagnosis represents the beginning rather than the culmination of adjustment for midlife and older autistic adults, and that meaningful change depends on the presence of specific social, relational, and informational supports.

### Diagnosis as Liminal: Recognition, Without Integration

Participants’ accounts strongly align with existing literature describing adult autism diagnosis as a liminal experience, a transitional state characterised by ambiguity, emotional upheaval, and lack of clear direction (Crane et al., 2020; Lewis, 2016). Diagnosis offered awareness of autism but often left individuals suspended between former understandings of the self and the unfamiliar self-identity of being autistic. For many, this liminality was compounded by shock, grief, or psychological distress, particularly among those who had internalised deficit-based narratives over the course of their lives.

Importantly, these findings should not be interpreted to suggest that diagnosis is unhelpful. Participants consistently valued receiving diagnostic confirmation. However, in the absence of structured post-diagnostic support, diagnosis often remained recognition without integration, a label situated within deficit-based narratives and lacking guidance on how to incorporate this new understanding into identity, relationships, and everyday life. This gap left several participants struggling emotionally and contributed to declining mental health for a subset of the sample. The liminal nature of diagnosis therefore reflects a failure of support systems rather than a failure of diagnosis itself. This systemic shortfall is echoed in a recent narrative synthesis by Norris et al. (2024), which demonstrates that autistic adults frequently express a desire for accessible, low-level support services following diagnosis, yet such provision remains inconsistent and limited across the United Kingdom.

### The Thriving Cycle as an Autistic Identity Development Trajectory

The grounded theory developed in this study, the ‘Thriving Cycle’, provides a conceptual explanation for why some midlife and older autistic adults thrive post-diagnosis while others remain in survival. The three interrelated processes identified—(a) acceptance and internalisation of autism, (b) exposure to positive constructions of autistic identity, and (c) ongoing self-reflection and knowledge acquisition—mirror established autistic identity development frameworks. Autistic identity theories propose that autistic people move through stages of awareness, exploration, internalisation, and belonging (Cage & Troxell-Whitman, 2019). Positive autistic identity is associated with reduced internalised stigma and improved well-being (Botha, 2021). The Thriving Cycle maps directly onto these, offering an empirically grounded model of how these processes unfold for midlife and older adults receiving a late-life diagnosis. This is consistent with quantitative and review evidence indicating that autistic identity is multidimensional and closely linked to well-being, particularly through processes of self-acceptance, social identification, and reduced internalised stigma (Cooper et al., 2021; Davies et al., 2024). While existing identity development frameworks are often described in stage-based terms, participants in this study did not report a uniform progression through clearly bounded stages. Instead, their accounts reflected a more fluid and recursive process. This interpretation is consistent with emerging evidence that post-diagnostic experiences involve oscillation between emotional states rather than progression through fixed stages (Mair et al., 2026), with the coexistence of distress, relief, and evolving self-understanding. While this study places greater emphasis on identity integration and agency, the convergence between these models strengthens the interpretation of post-diagnostic adjustment as dynamic and processual rather than fixed.

Thriving participants progressed beyond mere awareness of autism into acceptance and internalisation of autism and a deep exploration of what it means to be autistic. This identity work was facilitated by access to positive autistic narratives, supportive partners, or peers (many of whom were online contacts), and opportunities to reinterpret life experiences through a positive autistic lens. This emphasis on identity reappraisal is also reflected in recent work highlighting how late identification prompts reinterpretation of the self across past, present, and future contexts (Mair et al., 2026). Similarly, Lilley et al.’s (2022, 2023) oral history research with late-diagnosed autistic adults showed that post-diagnostic adjustment involves substantial autobiographical reappraisal, movement through negative self-conceptions, and gradual development of a more accepting autistic self, rather than immediate resolution following diagnosis (Lilley et al, 2022). Their later analysis (2023) further demonstrates that autistic identity is actively shaped through relationships, including family identifications and engagement with the autistic community, highlighting the importance of shared understanding and connection with other autistic people in developing a positive sense of self. Together, these studies reinforce the view that thriving emerges through ongoing identity work supported by affirming social contexts, rather than through diagnosis alone. In this study, participants described developing greater clarity, self-compassion, and self-efficacy as they moved through the cycle. However, it should be noted that for some this process took several years post-diagnosis to commence the ‘Thriving Cycle’.

In contrast, participants whose accounts reflected the surviving pattern remained at early, conflicted stages of identity development. Lacking opportunities to engage with positive autistic narratives, they continued to view autism through deficit-based frameworks, experienced chronic self-blame, and struggled to accept autism as part of their identity. The ‘Thriving Cycle’ thus helps explain divergent trajectories and highlights the central role of identity reconstruction in post-diagnostic well-being. This is consistent with research demonstrating that internalised stigma and deficit-based constructions of autism are associated with poorer psychological outcomes, whereas positive autistic identity and community connectedness are linked to improved well-being (Botha, 2021; Cooper et al., 2021).

### Why Diagnosis Alone is Not Enough

Situating these findings within identity development theory clarifies why diagnosis alone rarely improves well-being. Diagnosis offers awareness, but awareness is only the first stage of identity formation (Cage & Troxell-Whitman, 2019). Recent systematic and meta-synthesis work further supports this interpretation, demonstrating that diagnosis alone is rarely sufficient to improve well-being without access to ongoing support, identity development opportunities, and social connection (Kiehl et al., 2024; Nayyar et al., 2025). Without access to autistic communities, positive representations, and opportunities for autism knowledge acquisition and self-reflection, participants lacked the resources required to move towards true self-understanding and adaptation of their lives. This is supported by evidence that autistic community and shared identity play a central role in post-diagnostic adjustment, providing validation, belonging, and opportunities to reinterpret personal experiences in more positive and coherent ways (Lilley et al, 2022, 2023). Botha’s (2021) minority stress framework further suggests that autistic identity is formed within a social context shaped by stigma and invalidation; thus, without support, many autistic adults cannot progress beyond early stages of identity development.

For midlife and older autistic adults who have spent decades navigating misunderstanding, masking, or trauma without explanation, the identity work required post-diagnosis is extensive. Diagnosis alone does not resolve internalised stigma or rewrite entrenched narratives of difference. Instead, the post-diagnostic environment, its supports, discourses, and relational context determines whether individuals move towards thriving or remain merely surviving.

### Implications for Clinical Practice and Service Delivery

The findings have significant implications for the organisation of adult autism services in the United Kingdom. Autism assessment is resource-intensive, with NHS waiting lists stretching multiple years (Macdonald et al., 2022) and private assessments costing thousands of pounds (Jones et al., 2021). Yet many participants indicated that diagnosis alone offered minimal benefit without guidance on how to process, integrate, or act upon this new information.

While these findings are situated within the U.K. service context, many of the processes identified are likely to be relevant across settings where adults receive an autism diagnosis later in life. In particular, the challenges associated with delayed diagnosis, limited post-diagnostic support, and the need for identity reconstruction have been reported internationally (Kiehl et al., 2024; Lilley et al., 2022).

Providing diagnosis without support therefore represents questionable clinical and economic value. Participants’ accounts illustrate that, in the absence of structured post-diagnostic pathways, diagnosis risks becoming a missed opportunity, leaving individuals in distress rather than enabling meaningful change. These gaps are increasingly acknowledged in U.K. policy frameworks, which advocate early information provision, improved navigation of services, and identity-supportive, preventive approaches to adult autism care (All-Party Parliamentary Group on Autism [APPGA]/National Autistic Society [NAS], 2019; HM Government, 2021; NHS England, 2019; National Institute for Health and Care Excellence [NICE], 2021).

The present findings also provide a framework for understanding how such support might be effectively structured in practice. The ‘Thriving Cycle’ offers a process-based model that aligns closely with emerging autistic-led interventions. For example, autistic-led programmes combining psychoeducation and peer support have been shown to foster increased confidence, validation, and a more positive understanding of autism (Crane et al., 2023). These elements map directly onto the processes identified in the Thriving Cycle.

Specifically, psychoeducation may support the development of self-understanding and self-awareness, while peer interaction may facilitate connection with the autistic community and exposure to positive constructions of autistic identity. Together, these processes promote reflection, self-discovery, and the development of personalised life-management strategies. In this way, the ‘Thriving Cycle’ provides a conceptual framework through which autistic-led and co-produced interventions can be understood and further developed. Embedding such approaches within post-diagnostic pathways may help move support beyond information provision towards facilitating identity reconstruction, personal agency, and sustained improvements in well-being. More broadly, these findings reinforce the importance of ensuring that autistic adults have timely access to identity-affirming information, community connection, and opportunities for ongoing meaning-making throughout the post-diagnostic period, rather than relying on diagnostic confirmation alone. Taken together, the present findings converge with a growing international evidence base demonstrating that post-diagnostic outcomes are shaped not by diagnosis itself, but by access to these social, relational, and identity-affirming resources (Davies et al., 2024; Kiehl et al., 2024; Lilley et al., 2022, 2023; Mair et al., 2026).

### Strengths, Limitations, and Future Research

This study has several strengths, including the use of CGT, rich qualitative engagement with midlife and older autistic adults, and involvement from an autistic advisory group that enhanced interpretative rigour. However, there are limitations. The sample was self-selecting and predominantly White British, which may limit transferability to more diverse groups. Participants were relatively engaged with autism-related communities, potentially biasing experiences towards those with higher access to peer networks. Accounts were retrospective, shaped by time and ongoing meaning-making processes. In addition, trajectories of surviving and thriving were reconstructed from narratives rather than observed longitudinally. Future research should examine identity trajectories prospectively across diverse cultural contexts, explore diagnostic liminality across different life stages, and evaluate identity-focused and waiting-well interventions that operationalise the Thriving Cycle in clinical practice.

## Conclusion

This study demonstrates that, for midlife and older autistic adults, diagnosis is necessary but not sufficient to improve well-being. Rather than representing an endpoint, diagnosis initiated a process of identity reconstruction that only some participants were able to progress through. Those whose accounts reflected the thriving pattern engaged in an ongoing process of self-discovery, community connection, and the development of personal agency, conceptualised in this study as the ‘Thriving Cycle’. In contrast, many participants remained within the surviving pattern despite valuing their diagnosis, highlighting that diagnostic recognition alone was insufficient to facilitate meaningful change. These findings provide empirical support for embedding diagnosis within broader, identity-focused post-diagnostic pathways that promote identity reconstruction, foster community connection, and support the development of personal agency. Supporting autistic adults to move beyond surviving towards thriving requires investment not only in timely diagnosis, but also in the relational, informational, and psychosocial resources that enable positive autistic identity reconstruction and sustained well-being.

## Data Availability

The qualitative datasets generated and analysed during the current study are not publicly available due to the sensitive and potentially identifiable nature of the interview data involving a small sample of older autistic adults. Participants did not provide consent for full transcript sharing, and public release may risk deductive disclosure despite anonymisation.

## Appendix

### Semi-Structured Interview Schedule

Interviews were conversational and participant-led, with prompts used flexibly to explore issues raised by participants. The schedule was refined iteratively throughout data collection.

#### Opening and Demographic Information

Participants first provided demographic information relevant to the study.

Participants were informed that interviews were conversational and that they could guide the order and depth of discussion.

1. Life History and Background
  - Childhood and school experiences
  - Experiences of difference and belonging
  - Family relationships
  - Education and employment
  - Relationships, friendships, and interests
2. Diagnosis and Meaning-Making
  - What led you to seek, or receive, an autism diagnosis?
  - What was it like receiving a diagnosis later in life?
  - What does the diagnosis mean to you now?
  - Has diagnosis changed how you understand yourself or your life experiences?
  - Do you disclose your diagnosis or autistic identity to others?
3. Well-Being and Mental Health

Participants were invited to reflect on factors influencing well-being across the life course.

- What kinds of experiences or environments negatively affect well-being?
- What kinds of experiences, relationships, or environments support well-being?
- What helps you feel calm, balanced, or emotionally well?
- What contributes to stress, anxiety, exhaustion, or overwhelm?
- What supports or changes improve quality of life?

4. Social Experiences and Relationships
  - What have friendships and social relationships been like for you?
  - How do you experience social interaction?
5. Self, Identity, and Strengths
  - What do you see as your strengths?
  - What do you see as your challenges?
  - How has your understanding of yourself changed over time?
6. Coping Strategies and Adaptation
  - What strategies or routines help support your well-being?
  - What have you learned about yourself over your life course?
  - Have your coping strategies changed over time or since diagnosis?
7. Services, Support, and Ageing
  - What kinds of support have been helpful to you?

#### Closing

- Is there anything important that we have not discussed, or that you would like professionals to better understand about autistic adults diagnosed later in life?

## References

[bibr1-13623613261483304] All-Party Parliamentary Group on Autism/National Autistic Society. (2019). The Autism Act, 10 years on: A report from the All-Party Parliamentary Group on Autism.

[bibr2-13623613261483304] Bargiela S. Steward R. Mandy W. (2016). The experiences of late-diagnosed women with autism spectrum conditions: An investigation of the female autism phenotype. Journal of Autism and Developmental Disorders, 46(10), 3281–3294. 10.1007/s10803-016-2872-8 PMC5040731 27457364

[bibr3-13623613261483304] Botha M. (2021). Autistic community connectedness as a protective factor: A grounded theory of belonging. Journal of Autism and Developmental Disorders, 51, 4179–4193.

[bibr4-13623613261483304] Botha M. Frost D. M. (2020). Extending the minority stress model to understand mental health problems experienced by the autistic community. Society and Mental Health, 10(1), 20–34.

[bibr5-13623613261483304] Botha M. Gillespie-Lynch K. (2022). Come as you are: Examining autistic identity development and the neurodiversity movement. Autism, 26(6), 1433–1436.

[bibr6-13623613261483304] Cage E. Troxell-Whitman Z. (2019). Understanding the identity experiences of autistic adults: A qualitative study. Journal of Autism and Developmental Disorders, 49(2), 442–455. 10.1007/s10803-018-03878-x PMC6483965 30627892

[bibr7-13623613261483304] Charmaz K. (2014). Constructing grounded theory (2nd ed.). Sage.

[bibr8-13623613261483304] Cooper R. Cooper K. Russell A. J. Smith L. G. E. (2021). “I’m proud to be a little bit different”: The effects of autistic individuals’ perceptions of autism and autism social identity on their collective self-esteem. Journal of Autism and Developmental Disorders, 51(2), 704–714. https://link.springer.com/article/10.1007/s10803-020-04575-4 10.1007/s10803-020-04575-4 PMC7835309 32607798

[bibr9-13623613261483304] Crane L. Adams F. Harper G. Welch J. Pellicano E. (2020). ‘I like that it’s so predictable’: Autistic adults’ experiences of assessment and diagnosis. Autism, 24(2), 442–457.

[bibr10-13623613261483304] Crane L. Hearst C. Ashworth M. Davies J. (2023). Evaluating the online delivery of an autistic-led programme to support newly diagnosed or identified autistic adults. Autism & Developmental Language Impairments, 8, 1–9. 10.1177/23969415231189608 PMC10387681 37529634

[bibr11-13623613261483304] Crane L. Maras K. Haworth K. Gibbs K. Kenny L. (2021). Experiences of autism diagnosis: A survey of over 1000 parents in the United Kingdom. Autism, 25(4), 1182–1196. 10.1177/1362361315573636 25810370

[bibr12-13623613261483304] Crowson S. Poole D. Scargill K. Freeth M. (2024). Understanding the post-diagnostic support priorities of autistic adults in the United Kingdom: A co-produced modified Delphi study. Autism, 28(4), 854–865. 10.1177/13623613231196805 PMC10981182 37776060

[bibr13-13623613261483304] Davies J. Cooper K. Killick E. Sam E. Healy M. Thompson G. Mallinday W. Redmayne B. Crane L. (2024). Autistic identity: A systematic review of quantitative research. Autism Research, 17(4), 873–893. 10.1002/aur.3105 38334318

[bibr14-13623613261483304] Higgins J. M. Arnold S. R. C. Weise J. Pelaez-Nogueras M. Pellicano E. (2021). “I felt like a faulty person”: Autistic adults’ experiences of the diagnostic process. Autism in Adulthood, 3(2), 147–157.

[bibr15-13623613261483304] HM Government. (2021). The autism strategy for England: 2021 to 2026.

[bibr16-13623613261483304] Hull L. Levy L. Lai M.-C. Petrides K. V. Mandy W. (2020). Autism, camouflaging and gender differences: A qualitative study. Molecular Autism, 11(1), 1–16.

[bibr17-13623613261483304] Hull L. Mandy W. Petrides K. V. (2017). Behavioural and cognitive sex/gender differences in autism spectrum condition and typically developing populations. Autism, 21(6), 706–727. 10.1177/1362361316669087 28749232

[bibr18-13623613261483304] Hull L. Petrides K. V. Allison C. Smith P. Baron-Cohen S. Lai M.-C. Mandy W. (2019). “Putting on my best normal”: Social camouflaging in adults with autism. Journal of Autism and Developmental Disorders, 49(2), 704–716. 10.1007/s10803-017-3166-5 PMC5509825 28527095

[bibr19-13623613261483304] Huws J. C. Jones R. S. P. (2010). ‘They just seem to live their lives in their own little world’: Lay perceptions of autism. Disability & Society, 25(3), 331–344.

[bibr20-13623613261483304] Jones D. R. English E. Guldberg K. Crane L. (2021). Early experiences of seeking an autism diagnosis in the United Kingdom: A qualitative study. Journal of Autism and Developmental Disorders, 51, 147–160.

[bibr21-13623613261483304] Kiehl I. Pease R. Hackmann C. (2024). The adult experience of being diagnosed with autism spectrum disorder: A qualitative meta-synthesis. Autism, 28(5), 1060–1073. 10.1177/13623613231230419 38360530

[bibr22-13623613261483304] Lai M.-C. Baron-Cohen S. (2015). Identifying the lost generation of autistic adults. The Lancet Psychiatry, 2(11), 1013–1027. 10.1016/S2215-0366(15)00277-1 26544750

[bibr23-13623613261483304] Leedham A. Thompson A. R. Smith R. Freeth M. (2020). “I was exhausted trying to figure it out”: The experiences of females receiving an autism diagnosis in adulthood. Autism, 24(1), 135–146. 10.1177/1362361319853442 31144507

[bibr24-13623613261483304] Lewis L. F. (2016). Exploring the experience of self-diagnosis of autism spectrum disorder in adults. Archives of Psychiatric Nursing, 30(5), 575–580. 10.1016/j.apnu.2016.03.009 27654240

[bibr25-13623613261483304] Lilley R. (2021). Autism, trauma, and identity: A qualitative exploration of post-diagnostic experiences. Autism in Adulthood, 3(1), 45–55.

[bibr26-13623613261483304] Lilley R. Lawson W. Hall G. Mahony J. Clapham H. Heyworth M. Arnold S. Trollor J. Yudell M. Pellicano E. (2023). “Peas in a pod”: Oral history reflections on autistic identity in family and community by late-diagnosed adults. Journal of Autism and Developmental Disorders, 53(3), 1146–1161. 10.1007/s10803-022-05667-z PMC9986211 35834048

[bibr27-13623613261483304] Lilley R. Lawson W. Hall G. Mahony J. Clapham H. Heyworth M. Arnold S. R. Trollor J. N. Yudell M. Pellicano E. (2022). ‘A way to be me’: Autobiographical reflections of autistic adults diagnosed in mid-to-late adulthood. Autism: The International Journal of Research and Practice, 26(6), 1395–1408. 10.1177/13623613211050694 34674564

[bibr28-13623613261483304] Lipinski S. Blanke E. Dziobek I. (2023). Late-diagnosed autism in adults: Predictors and psychosocial outcomes. Journal of Autism and Developmental Disorders, 53, 1230–1244.

[bibr29-13623613261483304] Livingston L. A. Shah P. Happé F. (2020). Compensatory strategies in autism: A qualitative study. Molecular Autism, 11(1), 1–17. 10.1186/s13229-019-0308-y PMC7014718 32051011

[bibr30-13623613261483304] Macdonald L. Clarke C. Adams D. (2022). Waiting for autism assessment: Families’ experiences and service implications. Autism, 26(4), 933–945.

[bibr31-13623613261483304] Mair A. P. A. Gonzalez-Figueroa M. McConachie D. Goodall K. Gillespie-Smith K. (2026). Grief, relief, and belief: A social media study on late identification of neurodivergence. Autism, 30(5), 1344–1359. 10.1177/13623613261437916 PMC13087149 41933478

[bibr32-13623613261483304] Mason D. McConachie H. Garland D. Petrou A. Rodgers J. Parr J. R. (2018). Predictors of quality of life for autistic adults. Autism Research, 11(8), 1138–1147. 10.1002/aur.1965 PMC6220831 29734506

[bibr33-13623613261483304] Milton D. Sims T. (2016). How is a sense of well-being and belonging constructed in the accounts of autistic adults? Disability & Society, 31(4), 520–534.

[bibr34-13623613261483304] National Autistic Society. (2022). The Autism Act: 12 years on report.

[bibr35-13623613261483304] National Institute for Health and Care Excellence. (2021). Autism spectrum disorder in adults: Diagnosis and management (NG142). 32186834

[bibr36-13623613261483304] Nayyar A. Debono J. Denman L. (2025). Lived experiences of adult autism diagnosis: A systematic review. Autism in Adulthood, 7(1), 1–12. doi: 10.1089/aut.2023.0152 PMC11937775 40151652

[bibr37-13623613261483304] NHS England. (2019). The NHS long term plan.

[bibr38-13623613261483304] Nicolaidis C. Raymaker D. Kapp S. K. Baggs A. Ashkenazy E. McDonald K. Weiner M. Maslak J. Hunter M. Joyce A. (2019). The AASPIRE practice-based guidelines for the inclusion of autistic adults in research as co-researchers and study participants. Autism, 23(8), 2007–2019. 10.1177/1362361319830523 PMC6776684 30939892

[bibr39-13623613261483304] Norris J. E. Harvey R. Hull L. (2024). Post-diagnostic support for adults diagnosed with autism in adulthood in the UK: A systematic review with narrative synthesis. Autism, 29(2), 284–309. 10.1177/13623613241273073 PMC11816465 39253875

[bibr40-13623613261483304] Pellicano E. den Houting J. (2022). Annual research review: Shifting from ‘normal science’ to neurodiversity in autism science. Journal of Child Psychology and Psychiatry, 63(4), 381–396. 10.1111/jcpp.13534 PMC9298391 34730840

[bibr41-13623613261483304] Russell G. Stapley S. Newlove-Delgado T. Salmon A. Ford T. (2021). Time trends in autism diagnosis over 20 years. Journal of Autism and Developmental Disorders, 51(2), 427–436.

[bibr42-13623613261483304] Stagg S. D. Belcher H. (2019). Living with autism without knowing: Receiving a diagnosis late in life. Health Psychology and Behavioral Medicine, 7(1), 348–361. 10.1080/21642850.2019.1684920 PMC8114403 34040855

[bibr43-13623613261483304] Tan C. (2018). Autistic adults’ experiences of identity, community, and support: A qualitative exploration. Autism in Adulthood, 1(1), 20–28.

[bibr44-13623613261483304] Wilson C. E. Roberts G. Gillan N. Brown K. S. Cobrin K. Murphy C. (2019). The lived experiences of autistic adults: A qualitative study. Autism, 23(2), 442–453.

